# Comparison of Antimicrobial Resistance among Commensal *Escherichia coli* Isolated from Retail Table Eggs Produced by Laying Hens from the Cage and Non-Cage Housing Systems in Western Australia

**DOI:** 10.3390/antibiotics12030588

**Published:** 2023-03-15

**Authors:** Hamid Reza Sodagari, Csaba Varga, Ihab Habib, Shafi Sahibzada

**Affiliations:** 1 Department of Pathobiology, College of Veterinary Medicine, University of Illinois Urbana-Champaign, Urbana, IL 61802, USA; 2 Carl R. Woese Institute for Genomic Biology, University of Illinois Urbana-Champaign, Urbana, IL 61802, USA; 3 Veterinary Public Health Research Laboratory, Department of Veterinary Medicine, College of Agriculture and Veterinary Medicine, United Arab Emirates University, Al Ain P.O. Box 1555, United Arab Emirates; 4 Department of Environmental Health, High Institute of Public Health, Alexandria University, Alexandria 21511, Egypt; 5 Antimicrobial Resistance and Infectious Diseases Laboratory, Murdoch University, Murdoch, WA 6150, Australia; 6 Australian Centre for Disease Preparedness, Commonwealth Scientific and Industrial Research Organization, Geelong, VIC 3220, Australia

**Keywords:** antibiotic resistance, multidrug resistance, *E. coli*, retail eggs, egg production system, Australia

## Abstract

Antimicrobial resistance (AMR) has become a global public health concern in recent decades. Although several investigations evaluated AMR in commensal and pathogenic bacteria from different foods of animal origin in Australia, there is a lack of studies that compared AMR in commensal *E. coli* isolated from retail table eggs obtained from different laying hen housing systems. This study aimed to determine AMR and differences in AMR patterns among *E. coli* isolates recovered from retail table eggs sourced from caged and non-caged housing systems in Western Australia. Commensal *E. coli* isolates were tested for susceptibility to 14 antimicrobials using a broth microdilution method. Clustering analyses and logistic regression models were applied to identify patterns and differences in AMR. Overall, there were moderate to high frequencies of resistance to the antimicrobials of lower importance used in Australian human medicine (tetracycline, ampicillin, trimethoprim, and sulfamethoxazole) in the isolates sourced from the eggs of two production systems. All *E. coli* isolates were susceptible to all critically important antimicrobials except the very low level of resistance to ciprofloxacin. *E. coli* isolates from eggs of non-caged systems had higher odds of resistance to tetracycline (OR = 5.76, *p* < 0.001) and ampicillin (OR = 3.42, *p* ≤ 0.01) compared to the isolates from eggs of caged systems. Moreover, the number of antimicrobials to which an *E. coli* isolate was resistant was significantly higher in table eggs from non-caged systems than isolates from caged systems’ eggs. Considering the conservative approach in using antimicrobials in the Australian layer flocks, our findings highlight the potential role of the environment or human-related factors in the dissemination and emergence of AMR in commensal *E. coli*, particularly in retail table eggs of non-cage system origin. Further comprehensive epidemiological studies are required to better understand the role of different egg production systems in the emergence and dissemination of AMR in commensal *E. coli*.

## 1. Introduction

Commercial layer farms play an essential role in providing protein sources by supplying eggs [1]. In Australia, the demand for the production and consumption of eggs is increasing. In the financial year 2021–2022, 6.6 billion eggs were produced by the Australian egg industry, and per capita egg consumption grew to 262 eggs, which was higher than in previous years [2].

Enteric bacteria can contaminate eggs from the colonized gut, the feces of layer hens during or after oviposition, or the infected reproductive organs with probable penetration into the eggshell [3,4]. *Escherichia coli* (*E. coli*) is a common commensal organism in humans and warm-blooded animals, such as birds and other livestock, with some opportunistic pathogen strains, which are responsible for a wide range of infections [5]. This organism is regarded as the optimal antimicrobial resistance (AMR) indicator in foods of animal origin [6] due to its ability to carry mobile genetic elements and other antibiotic resistance determinants that might transfer to other strains that reside in the host [7].

The widespread use of antimicrobials in food animal production, including poultry production, raises a significant global public health concern that is often associated with the emergence of resistance against antimicrobials that are commonly used in those animals [8,9,10]. Humans are exposed to resistant microorganisms through the consumption of raw or inadequately cooked eggs added to some desserts and sauces [11] or from cross-contamination during meal preparation [12], which promotes the risk of therapeutic failures if humans are infected with multidrug-resistant bacteria [13,14].

Australia practices a cautious regulatory approach concerning the use of critically important antimicrobials in food-producing animals. For instance, most critical antimicrobials including fluoroquinolones and gentamicin are restricted to being used in food animals. Moreover, the mass administration of third-generation cephalosporins and ceftiofur is limited in livestock and is also not approved for use in the poultry industry [15,16,17]. There is also minimal use of antimicrobials in the Australian commercial layer industry due to the risk of antimicrobial residues in eggs [15]. Only a few therapeutic antibiotics are permitted to be used in layer flocks, with a zero-withdrawal time for eggs, including chlortetracycline, a lincomycin-spectinomycin combination, as well as a brand of amoxicillin [18]. However, despite this admirable stewardship of antimicrobial use, there are still reports of AMR among commensal *E. coli* isolates in the egg production industry in Australia [19,20].

Different egg production systems, including cage, barn, and free-range, are utilized in commercial layer farms in Australia. Although each of these production systems has both advantages and disadvantages, the development of non-cage production systems (particularly free-range) in commercial layer farms has been driven by Australian consumer demand for reasons related to the birds’ welfare and the quality of eggs [21,22]. Free-range egg production has grown significantly over the last 15 years, and in 2020–2021, it comprised 52% of all retail grocery sales, which is higher than the cage system (36%) [2].

According to previous studies, the free-range egg production system appears to face more challenges related to biosecurity implementation over the control of environmental stressors and vectors [23,24]. The elevated environmental contact of free-range layer birds compared to caged ones may increase the incidence of infectious poultry diseases requiring treatment with antimicrobials [18], which might increase the emergence of resistant commensal and pathogenic bacteria. Therefore, we hypothesize that there might be differences in AMR patterns among commensal *E. coli* isolated from retail table eggs obtained from the cage and non-cage housing systems.

Differences in AMR determinants in *E. coli* isolates were previously investigated in different bird species and housing systems in Canada [25] as well as in laying hens and eggs in conventional and organic keeping systems in Germany [26]. Although limited studies have investigated AMR in commensal *E. coli* from table eggs [19] and layer farm environments in Australia [20], to the best of our knowledge, no published literature has compared the AMR patterns of *E. coli* isolates sourced from retail table eggs of the cage and non-cage housing systems. Therefore, to address this knowledge gap, this study has aimed to determine the prevalence of AMR and multidrug resistance (MDR) in *E. coli* isolated from retail table eggs of caged and non-caged origin and assess the association between resistance carriage in *E. coli* with layer housing systems in Western Australia.

## 2. Results

### 2.1. Description of Submissions

A total of 100 *E. coli* isolates recovered from retail table egg samples of the cage and non-cage origin were selected for antimicrobial susceptibility testing. We combined the isolates recovered from table eggs produced by the barn and free-range housing systems as “non-cage” (*n* = 68) to enable better statistical power when comparing with the isolates obtained from table eggs sourced from the cage housing system (*n* = 32).

### 2.2. Descriptive Analyses

Analysis indicated that 57 (57%) of the 100 *E. coli* isolates were resistant to at least one antimicrobial agent. The highest resistances observed were to tetracycline (*n* = 49, 49%), followed by ampicillin (*n* = 36, 36%), trimethoprim (*n* = 20, 20%), sulfamethoxazole (*n* = 18, 18%), ciprofloxacin (*n* = 2, 2%), and tigecycline (*n* = 1, 1%). Data indicated that all the isolates were susceptible to azithromycin, meropenem, cefotaxime, ceftazidime, chloramphenicol, nalidixic acid, colistin, and gentamicin (Table 1).

In the *E. coli* isolates obtained from the eggs of the cage system, there was a moderate prevalence of resistance (15–39%) to tetracycline and ampicillin, a low frequency of resistance (5–14%) to trimethoprim and sulfamethoxazole, and a very low prevalence of resistance (<5%) to ciprofloxacin (Table 1).

In the non-cage table egg origin *E. coli* isolates, there was a high prevalence of resistance (≥40%) to tetracycline and ampicillin, a moderate prevalence of resistance (15–39%) to trimethoprim and sulfamethoxazole, and a very low prevalence of resistance (<5%) to ciprofloxacin and tigecycline (Table 1).

Multidrug resistance was detected in 9.4% (95% CI = 1.9–25%) of the *E. coli* isolates from the eggs of cage housing systems and 26.5% (95% CI = 16.5–38.6%) of the isolates from the eggs of non-cage system. The AMR and MDR patterns of *E. coli* isolates are shown in Table 2. Two and four MDR profiles (microbiologically resistant to three or more classes of antimicrobials) were identified in the *E. coli* isolates recovered from retail table eggs of the cage and non-cage housing systems, respectively. Our findings indicated that the most common MDR pattern in the isolates of both caged (2 isolates) and non-caged (13 isolates) source retail table eggs was the concurrent resistance to sulfamethoxazole-trimethoprim-ampicillin-tetracycline (Table 2).

**Figure 1:**
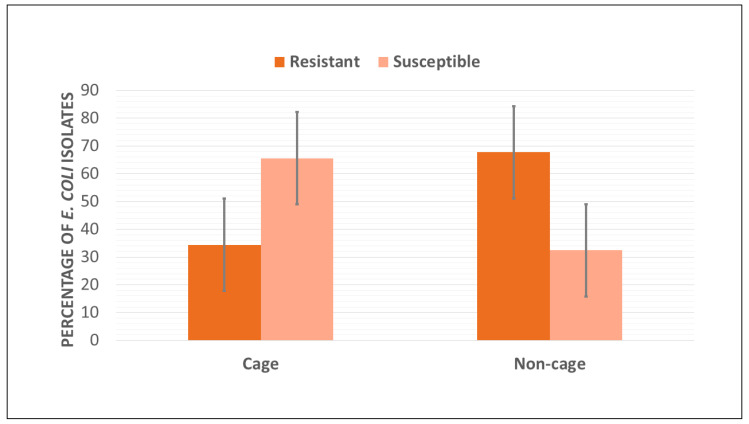
Percentage of resistant and susceptible *E. coli* isolates (*n* = 100) recovered from retail table eggs sourced from cage and non-cage housing systems in Western Australia.

It is worth mentioning that no resistance to the 14 antimicrobials tested was identified in 43% of the *E. coli* isolates. Isolates sourced from retail table eggs of the cage systems showed a higher rate of susceptibility (65.6%) compared to the isolates recovered from table eggs of the non-cage housing systems (32.3%) (Figure 1).

### 2.3. Cluster Analyses

**Figure 2:**
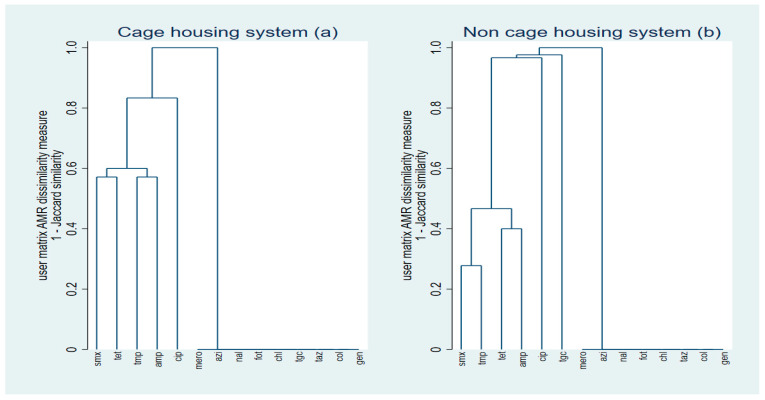
Single-linkage clustering dendrograms of resistance of egg-sourced *E. coli* (*n* = 100) isolates to antimicrobials by production system ((**a**) cage and (**b**) non-cage). SMX: sulfamethoxazole; TMP: trimethoprim ampicillin; CIP: ciprofloxacin; NAL: nalidixic acid; TET: tetracycline; MERO: meropenem; AZI: azithromycin; FOT: cefotaxime; TAZ: ceftazidime; CHL: chloramphenicol; TGC: tigecycline; COL: colistin; AMP: ampicillin; GEN: gentamicin.

Single-linkage clustering dendrograms with Jaccard distances for *E. coli* resistance are represented in Figure 2. In the dendrogram showing the *E. coli* isolates sourced from the cage system, three main resistance clustering patterns were identified, the patterns of resistance to sulfamethoxazole and tetracycline, trimethoprim and ampicillin, and a cluster of isolates that were susceptible to azithromycin, meropenem, cefotaxime, ceftazidime, chloramphenicol, nalidixic acid, colistin, tigecycline, and gentamicin (Figure 2a). The dendrogram of the *E. coli* isolates from retail table eggs of non-cage systems also included three main resistance clustering patterns. One cluster showed resistance to sulfamethoxazole and trimethoprim, whereas the other showed resistance to ampicillin and tetracycline. A relatively high proportion (i.e., a cluster) of the isolates were also susceptible to azithromycin, meropenem, cefotaxime, ceftazidime, chloramphenicol, nalidixic acid, colistin, and gentamicin (Figure 2b).

**Figure 3:**
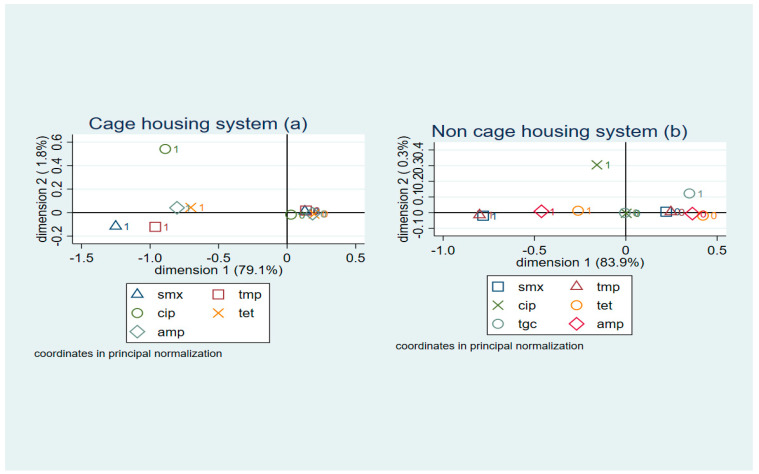
Multiple correspondence analysis coordinate plot displaying the presence (1) and absence (0) of resistance to six antimicrobials in *E. coli* isolates (*n* = 100) from (**a**) cage and (**b**) non-cage retail table eggs for the first two dimensions. SMX: sulfamethoxazole; TMP: trimethoprim; CIP: ciprofloxacin; TET: tetracycline; TGC: tigecycline; AMP: ampicillin.

Multiple correspondence analysis coordinate plots for the first two dimensions of resistance in *E. coli* isolates from retail egg samples of the cage and non-cage systems are shown in Figure 3. Eight antimicrobials (azithromycin, cefotaxime, ceftazidime, chloramphenicol, meropenem, nalidixic acid, colistin, and gentamicin) were removed from the analysis because not enough variation was identified. For *E. coli* isolates from eggs of the caged housing system, the first two dimensions explained 79.1% of the variation in antimicrobial resistance (Figure 3a). A high relatedness of resistance to ampicillin and tetracycline (co-resistance cluster) was observed, and a lower degree of relatedness between resistance to trimethoprim and sulfamethoxazole was also noted.

For *E. coli* isolates obtained from eggs of non-caged systems, the first two dimensions explained 83.9% of the variation in AMR (Figure 3b), and a cluster of co-resistance to trimethoprim and sulfamethoxazole was detected (when observation scores were plotted along dimensions 1 and 2). A lower degree of relatedness between resistance to ampicillin and tetracycline was also identified.

### 2.4. Logistic Regression

The odds of resistance to tetracycline (Odds Ratio = 5.76, 95% CI = 2.18–15.22, *p* < 0.001) were almost 6 times higher, and the odds of resistance to ampicillin were 3.4 times higher (OR = 3.42, 95% CI = 1.24–9.37, *p* = 0.017) in *E. coli* isolates sourced from retail table eggs of non-caged housing systems compared to the isolates sourced from the egg samples of the caged system.

### 2.5. Poisson Regression

The number of antimicrobials to which an *E. coli* isolate was resistant was significantly higher (incidence rate ratio = 2.27, 95% CI = 1.39–3.73, *p* = 0.001) in the isolates from eggs originating from non-caged housing systems when compared to the isolates from table egg samples of caged system source.

## 3. Discussion

The present study evaluated AMR in commensal *E. coli* isolates originating from caged and non-caged retail table eggs in Western Australia, representing a relatively moderate to high frequency of resistance to antimicrobials commonly used to treat bacterial infections in poultry and other livestock. Resistance to the critically important antimicrobials in human medicine was rare among *E. coli* isolated from retail table eggs in Western Australia. This study identified a higher resistance carriage to some of the antimicrobials in *E. coli* isolated from table eggs from the non-cage system when compared to the cage system. Our findings have provided helpful baseline data that will promote our understanding of AMR in commensal *E. coli* originating from retail table eggs sourced from caged and non-caged systems in Western Australia. These findings might aid in achieving “objective three” of Australia’s first national antimicrobial resistance strategy to enhance the nationally coordinated One Health surveillance of antimicrobial resistance and antimicrobial usage.

Given the differences in sampling settings and protocols and the lab and analytical methods employed, direct comparisons between the present study and similar previous investigations should be treated with caution. Our findings indicated high to moderate resistance among *E. coli* isolates to historically used antimicrobials that are still used in animal production [27], including tetracycline (49%), ampicillin (36%), trimethoprim (20%), and sulfisoxazole (18%). In Australia, *E. coli* isolates from the commercial egg industry have been identified as being highly resistant to the same antimicrobials [20]. Although low levels of resistance to aminoglycoside (1%) and phenicol (2.4%) were reported in the study mentioned above in Australia, no *E. coli* isolates were resistant to the same classes of antimicrobials in the present investigation. Despite different sampling settings and frameworks, farm-related factors such as antimicrobial use and health management may play an important role in the variations in the AMR prevalence of commensal *E. coli* isolates in different studies.

According to our results, the resistance of *E. coli* isolates recovered from non-caged origin retail table eggs to historically used antimicrobials in poultry production, including tetracycline, ampicillin, trimethoprim, and sulfisoxazole, was considerably higher than the prevalence of resistance to the same antimicrobials in *E. coli* isolates sourced from egg samples from the caged system.

Previous investigations have demonstrated that when comparing different housing systems, it is evident that freshly laid eggs in the cage housing system have a lower bacterial load than eggs from non-cage housing systems [28,29,30,31]. This could be due to the higher contact of eggs in the non-cage system with a contaminated environment or perhaps due to the higher shedding of bacteria in non-caged layer birds compared to caged birds because of the higher prevalence of environmental stressors [23]. It could be hypothesized that the higher level of bacterial contamination of eggs from the non-caged system might be associated with a higher level of resistant bacteria on the eggs. Further studies at the farm and retail levels are required to confirm or disprove this hypothesis.

On the other hand, our findings might reinforce the previous hypothesis mentioned, in the commercial egg production industry, shifting towards more extensive production systems (i.e., free-range) has promoted the incidence of many diseases, resulting in a return to the use of medications [18]. This might increase the emergence of resistant bacteria in such a housing system, which warrants further comprehensive research. Nonetheless, different levels of biosecurity measures at farms and human-related factors at the retail level also cannot be ignored in relation to the emergence and dissemination of resistant bacteria to humans through the consumption of eggs or egg products. Since Australian egg layers generally discourage the prophylactic use of antimicrobials, more strict levels of biosecurity might be required in the future due to the increasing number of higher-welfare management systems, including free-range and barn systems.

Among the critically important antimicrobials tested in the present study, only a very low level of resistance to ciprofloxacin was identified in *E. coli* isolates from table eggs sourced from both cage (3.1%) and non-cage (1.5%) systems. However, without complete plasmid DNA sequencing, it cannot be fully concluded that these few isolates were resistant to fluoroquinolone [19]. These results concur with the previous studies, which reported rare resistance to critically important antimicrobials in *E. coli* isolated from Australian livestock, including from commercial egg layer farms [20], meat chickens [32], pigs [33], and cattle [34]. Our findings, although promising, further highlight the potential role of environmental or human-related factors in detecting non-wild type ciprofloxacin-resistant *E. coli* in the absence of local antimicrobial selection pressure at layer farms in Australia [19]. Future investigations might be necessary to prove this hypothesis.

The absence of resistance to nearly all of the critically important antimicrobials in our investigation could be due to the presence of a conservative approach in the general use of these antimicrobials in food-producing animals [35], particularly in the commercial egg layer industry [15] in Australia. Based on these promising findings, it is hypothesized that commensal *E. coli* isolates originating from table eggs sourced from both cage and non-cage systems in Western Australia will continue to be susceptible to critical antimicrobials in human medicine in the future. However, continuous AMR surveillance of table eggs and environmental samples from Western Australia’s egg industry will shed further light on this hypothesis.

Four MDR profiles were identified in the present study, and the most common MDR pattern in *E. coli* isolates was co-resistance to three classes of antimicrobials, including beta-lactams, folate pathway inhibitors, and tetracycline. Concurrent resistance to the same antimicrobial classes was also recently reported for the MDR *E. coli* isolates from the Australian commercial egg industry [20]. Our findings are encouraging when compared to the previous study [20], wherein the presence of a few *E. coli* isolates resistant to four and five antimicrobial classes was also reported, which was not identified in the present study. In our investigation, the higher frequency of MDR in *E. coli* isolated from table eggs of non-caged systems (26.5%) compared to the *E. coli* isolated from table eggs of caged systems (9.4%) might highlight the critical role of environmental vectors and stressors in the non-caged egg production system. However, more comprehensive and comparative studies might be needed to confirm our findings and to investigate other effective factors related to the role of housing systems in the development of MDR in commensal *E. coli* in Western Australia’s commercial egg industry.

Our single-linkage clustering analysis of the *E. coli* isolates sourced from non-caged retail eggs demonstrated concurrent resistance to sulfamethoxazole and trimethoprim as well as tetracycline and ampicillin. Antimicrobial resistance clusters of *E. coli* isolates sourced from non-caged retail eggs also consisted of the same antimicrobials, including a cluster of co-resistance to sulfamethoxazole and tetracycline and another cluster of concurrent resistance to trimethoprim and ampicillin, which are widely used antibiotics in human and veterinary medicine. The results also indicated that almost all of the *E. coli* isolates recovered from the eggs produced by both housing systems were pan-susceptible to the critically important antimicrobials in human medicine, which is an encouraging and expected finding because of the minimal use of these antibiotics in the Australian commercial layers [18]. Our MCA analysis for the *E. coli* isolates originating from table eggs samples from cage and non-cage systems indicated a high degree of relatedness (e.g., co-resistance) between resistance to folate pathway inhibitors antimicrobials, including sulfamethoxazole and trimethoprim. Similar resistance mechanisms between these two members of the folate pathway inhibitors antimicrobial class [36] might be the reason for this high relatedness. A lower degree of relatedness between resistance to ampicillin and tetracycline was also identified. Our MCA findings for the *E. coli* isolates sourced from table eggs of the caged housing system were slightly different from our single-linkage clustering, possibly due to the differences between these two clustering methods.

The concurrent resistance of the retail egg *E. coli* isolates to the commonly used antimicrobials in food animal production highlights the necessity of the judicious use of these antimicrobials in food-producing animals to reduce the development and dissemination of AMR and MDR bacteria at the farm level [37,38]. It is important to highlight that there might be a higher probability of intestinal colonization with resistant bacterial strains than with susceptible strains; however, the underlying reasons for this phenomenon must be investigated by further research. It is also worth mentioning that the absence of relatedness between resistance to quinolones and the other antimicrobial classes is an encouraging finding of the present study. Nonetheless, continuous monitoring through an effective ongoing AMR surveillance program at farms and the retail level is required to maintain the minimum level of quinolone-resistant *E. coli* in Western Australia’s egg industry.

Our regression models indicated a higher probability of resistance to tetracycline and ampicillin among the *E. coli* isolates from non-caged produced retail eggs compared to the isolates recovered from eggs of caged systems. The results also indicated that the number of antimicrobials to which an *E. coli* isolates was resistant was significantly higher in E.coli isolates from the eggs of non-caged systems when compared to the isolates from eggs of the caged system. Differences in AMR between the two main egg production systems (cage and non-cage) might be partly explained by the variations in antimicrobial use and husbandry practices at the cage and non-cage layer farms. Moreover, the role of environmental and human-related factors from farms to the retail stage cannot be ignored. Both hypotheses underscore the need for further research to verify our findings and investigate other related factors in the development of AMR and MDR in commensal *E. coli* in foods of animal origins.

The limitations of this study include sampling bias, as free-range eggs were purposively oversampled because of their growing demand in Australia; therefore, the higher frequency of *E. coli* isolates recovered from non-caged (68%) compared to caged eggs (32%), as well as the low overall number of tested *E. coli* isolates (*n* = 100) in this study, might have influenced the AMR prevalence of commensal *E. coli* in both cage and non-cage retail table egg samples.

## 4. Materials and Methods

### 4.1. Study Design and Lab Methods

The study design and laboratory methods were described in detail in our previously published study [19]. Briefly, a total of 181 retail egg samples (each containing one dozen eggs) collected from different supermarkets across Perth (Western Australia) were tested for the isolation of commensal *E. coli* using the ISO 16649-1:2018 standard [39]. The selected colonies were then confirmed to species level using MALDI-TOF (matrix-assisted laser desorption ionization time-of-flight) with research-use-only (RUO) library databases, version claim 4 (microflex instrument, Bruker Diagnostics, Germany).

Antimicrobial susceptibility of confirmed *E. coli* isolates (*n* =100) to 14 antimicrobials was conducted in a micro-broth dilution using commercially prepared panels (Sensititre EUVSEC, TREK Diagnostic Systems, Thermo Fisher Scientific) according to the manufacturer’s guidelines, and quality control strains *E. coli* ATCC25922 were used throughout the testing. Minimum inhibitory concentrations (MICs) were interpreted using microbiological cut-off values (also referred to as ‘Epidemiological Cut-off Values’ or ECOFFs). It is worth mentioning that ECOFFs are not a predictor of clinical success but rather measures of an antimicrobial drug MIC distribution that separate bacterial populations into microbiologically susceptible (wild type) and microbiologically resistant (non-wild type) [20]. In the present investigation, we used ECOFFs values represented by the European Committee on Antimicrobial Susceptibility Testing (EUCAST) [40]. Isolates that are microbiologically resistant to three or more antimicrobials classes based on ECOFFs are classified as MDR phenotype [20]. To simplify the explanation of individual and multidrug resistance patterns, we used the words “susceptible” and “resistant” for microbiologically susceptible (wild type) and microbiologically resistant (non-wild type) isolates, respectively.

### 4.2. Data Analysis

Antimicrobial susceptibility data were transferred into a spreadsheet in Microsoft Excel (Microsoft Office 2016), checked for missing values, and subsequently imported into a statistical software program (STATA Intercooled software Version 17, Stata Corporation, College Station, TX, USA) for analysis.

#### 4.2.1. Descriptive Analysis

For egg samples from caged and non-caged housing systems, estimates of the proportion of *E. coli* isolates resistant to each tested antimicrobial were determined by dividing the number of isolates resistant to an antimicrobial by the total number of isolates tested for the antimicrobial. Added to that, estimates of the proportion of isolates that demonstrated MDR were calculated by dividing the number of MDR isolates by the total number of tested isolates. It should be noted that a sample was considered to be resistant to an antimicrobial if at least one isolate from that sample was resistant to that antimicrobial. Confidence intervals (CIs) were computed using exact binomial CIs using the Clopper–Pearson method for all proportions.

#### 4.2.2. Cluster Analysis

Cluster analysis, using the Jaccard binary similarity coefficient, was conducted for each variable (eggs from cage and non-cage systems) to compare individual antimicrobials concerning their similarity in the resistance status of *E. coli*. Dendrograms were constructed using the single-linkage clustering method with the Jaccard distance. The dissimilarity between antimicrobials was measured by Jaccard distance by subtracting the Jaccard binary similarity coefficient from one [41]. Therefore, a high dissimilarity measure shows that relatively few isolates were resistant to both antimicrobials. In contrast, a low dissimilarity measure indicates a relatively high proportion of isolates resistant to both antimicrobials. All isolates were considered susceptible to both antimicrobials when the dissimilarity measure was zero.

Multiple correspondence analysis, using the Burt method with principal normalization [42,43], was constructed for *E. coli* isolates recovered from table egg samples produced by the cage and non-cage housing systems to identify relationships within the set of six selected antimicrobials in terms of their similarity in *E. coli* resistance. We included dimensions that explained at least two-thirds of the variation in the data for further analysis. Observation scores were computed and plotted to visualize the distribution of AMR patterns along the first two dimensions.

#### 4.2.3. Logistic Regression

To identify differences in *E. coli* resistance between housing systems (cage and non-cage), logistic regression was applied; only antimicrobials for which ≥5% of the isolates were resistant were evaluated. Therefore, 4 of 14 antimicrobials were analyzed, including sulfamethoxazole, trimethoprim, ampicillin, and tetracycline. One logistic regression model was made for each antimicrobial. In these univariable models, the dependent variable indicated the prevalence of resistance to the antimicrobial, whereas the independent variable was the production systems (non-cage compared to cage). A *p*-value ≤ 0.05 on the Wald χ^2^ test demonstrated a statistically significant association.

Added to that, Poisson regression models were built to identify differences in *E. coli* MDR between the egg samples produced by two production systems (cage and non-cage). In this model, the independent variable was the production system (cage and non-cage), and the dependent variable was the number of antimicrobials to which an isolate was resistant.

## 5. Conclusions

Our findings demonstrated moderate to high levels of resistance to antimicrobials of lower importance in human medicine in the *E. coli* isolates from eggs produced by both housing systems. An exceptionally low level of resistance to critically important antimicrobials (ciprofloxacin) in human medicine was also detected in a few *E. coli* isolates from the eggs produced by both cage and non-cage systems. In this study, the higher prevalence of AMR and MDR in *E. coli* isolates recovered from eggs sourced from the non-caged housing systems compared to the isolates recovered from eggs of the caged system highlights the necessity of further research regarding the early detection of AMR in commensal and pathogenic bacteria, specifically in the non-caged egg production system, which is the popular system viewed by the public due to its higher welfare status of laying hens. Further comprehensive epidemiological studies are required to better understand the role of different egg production systems in the emergence and dissemination of AMR in commensal *E. coli* to meet expectations regarding the safety of food products and humans in Australia’s future.

## Figures and Tables

**Table 1:** Number and percentage of commensal *E. coli* isolates (*n* = 100) from Western Australian retail table eggs of caged and non-caged origin that were resistant to 14 selected antimicrobials.

Antimicrobial Class	Antimicrobial ^A^	Cage (*n* = 32) *n* (%) ^B^ [CI] ^C^	Non-Cage (*n* = 68) *n* (%) ^B^ [CI] ^C^	Total (*n* = 100) *n* (%) ^B^ [CI] ^C^
Folate pathway inhibitors	SMX	3 (9.4) [2–25]	15 (22) [12.9–33.7]	18 (18) [11–26.9]
	TMP	4 (12.5) [3.5–29]	16 (23.5) [14.1–35.4]	20 (20) [12.7–29.2]
Quinolones	CIP	1 (3.1) [0.08–16.2]	1 (1.5) [0.04–7.92]	2 (2) [0.2–7]
	NAL	0	0	0
Tetracyclines	TET	7 (21.9) [9.28–40]	42 (61.8) [49.2–73.3]	49 (49) [38.9–59.2]
Carbapenem	MERO	0	0	0
Macrolides ^D^	AZI	0	0	0
Third-generation cephalosporin	FOT	0	0	0
	TAZ	0	0	0
Phenicols	CHL	0	0	0
Glycylcyclines	TGC	0	1 (1.5) [0.04–7.92]	1 (1) [0–5.4]
Polymixins	COL	0	0	0
Beta-lactams	AMP	6 (18.7) [7.2–36.4]	30 (44.1) [32.1–56.7]	36 (36) [26.6–46.2]
Aminoglycosides	GEN	0	0	0

^A^ SMX: sulfamethoxazole; TMP: trimethoprim ampicillin; CIP: ciprofloxacin; NAL: nalidixic acid; TET: tetracycline; MERO: meropenem; AZI: azithromycin; FOT: cefotaxime; TAZ: ceftazidime; CHL: chloramphenicol; TGC: tigecycline; COL: colistin; AMP: ampicillin; GEN: gentamicin. ^B^ Number and percentage of isolates resistant to the antimicrobial. ^C^ CI = Exact binomial 95% confidence interval for the percentage of isolates resistant to the antimicrobial. ^D^ No ECOFF was available for the combination *E. coli*/Azithromycin.

**Table 2:** Antimicrobial resistance patterns of commensal *E. coli* (*n* = 100) isolated from retail table eggs of the cage and non-cage housing systems in Western Australia.

Production System	Antimicrobial Resistance Pattern ^A^	Number of Antimicrobial Classes in Pattern (Multidrug-Resistant) ^B^	*n* (%) ^C^
Cage	TET	1 (no)	3 (9.37)
	TMP	1 (no)	1 (3.12)
	AMP	1 (no)	2 (6.25)
	TMP-AMP	2 (no)	1 (3.12)
	SMX-TET	2 (no)	1 (3.12)
	CIP-TET-AMP	3 (yes)	1 (3.12)
	SMX-TMP-TET-AMP	3 (yes)	2 (6.25)
Non-cage	AMP	1 (no)	3 (4.41)
	TET	1 (no)	14 (20.58)
	SMX	1 (no)	1 (1.47)
	TET-AMP	2 (no)	9 (13.23)
	TET-TGC	2 (no)	1 (1.47)
	TMP-TET-AMP	3 (yes)	3 (4.41)
	SMX-TET-AMP	3 (yes)	1 (1.47)
	CIP-TET-AMP	3 (yes)	1 (1.47)
	SMX-TMP-TET-AMP	3 (yes)	13 (19.11)

^A^ SMX: sulfamethoxazole; TMP: trimethoprim ampicillin; CIP: ciprofloxacin; TET: tetracycline; TGC: tigecycline; AMP: ampicillin. ^B^ An isolate was defined as multidrug-resistant if it was resistant to at least one antimicrobial in ≥3 antimicrobial classes. ^C^ Number and percentage of isolates belonged to each antimicrobial resistance pattern.

## Data Availability

Not applicable.
